# Low T3 syndrome is associated with the severity of myelin oligodendrocyte glycoprotein antibody-associated disease exacerbation

**DOI:** 10.3389/fnins.2024.1357633

**Published:** 2024-05-21

**Authors:** Yuqing Wu, Hao Zhou, Xiaojiao Ci, Jie Lu

**Affiliations:** Department of Neurology, The Affiliated Brain Hospital of Nanjing Medical University, Nanjing, Jiangsu, China

**Keywords:** myelin oligodendrocyte glycoprotein antibody-associated disease, thyroid dysfunction, anti-thyroid antibody, low T3 syndrome, expanded disability status scale

## Abstract

**Background:**

Myelin oligodendrocyte glycoprotein antibody-associated disease (MOGAD) is a rare autoimmune inflammatory disease of the central nervous system, (CNS) different from multiple sclerosis (MS) and neuromyelitis optica spectrum disorder (NMOSD). While numerous studies have delved into the involvement of thyroid antibodies (ATAbs) and thyroid function in NMOSD and MS. The objective of this study is to explore the clinical significance of thyroid dysfunction and ATAbs abnormalities in adult patients with MOGAD.

**Methods:**

36 adult inpatients diagnosed with MOGAD and 47 sex- and age-matched healthy controls were enrolled. Patients were divided into two groups based on the presence or absence of low T3 syndrome. Demographics, clinical characteristics, and results of auxiliary examinations were compared across the subgroups. Moreover, an analysis was conducted to explore the correlations between thyroid hormone levels and Expanded Disability Status Scale (EDSS) scores.

**Results:**

Thyroid dysfunction was notably more frequent in MOGAD patients than healthy controls (*p* < 0.0001), particularly low T3 syndrome (*p*=0.03). Furthermore, subgroup analyses revealed that the low T3 syndrome group exhibited higher EDSS scores and a higher proportion of individuals with EDSS scores > 3, in comparison to the non-low T3 syndrome group (*p* = 0.014, *p* = 0.046). However, no significant differences were observed in demographic characteristics, annual relapse rates, clinical phenotypes, laboratory and MRI results, and EEG abnormalities between the two groups. Additional Spearman's analysis showed significantly negative correlations between the TT3 and FT3 levels with EDSS scores (r = −0.367, *p* = 0.028; r = −0.377, *p* = 0.024). Typical brain lesions and paralateral ventricle lesions were significantly rare in patients with positive ATAbs compared to those with negative ATAbs (*p* = 0.0001, *p* = 0.03), although the incidence of ATAbs abnormalities did not differ significantly between MOGAD patients and healthy controls.

**Conclusions:**

Overall, this study confirmed thyroid dysfunction, especially low T3 syndrome, is frequent in adult MOGAD patients. Patients with low T3 syndrome exhibited elevated EDSS scores and a significantly higher incidence of unfavorable condition. additionally, the correlation analysis model manifests that FT3 and TT3 levels were negatively correlated with EDSS scores. These evidences indicate that low T3 syndrome is associated with the severity of MOGAD exacerbation.

## 1 Introduction

Myelin oligodendrocyte glycoprotein (MOG), a glycoprotein that is highly conserved among species and uniquely expressed in oligodendrocytes of the mammalian central nervous system (CNS) (Lebar et al., 1989). Masses of experimental models of inflammatory demyelinating diseases have identified that MOG is an encephalitogentic protein capable of triggering a demyelinating immune response (Iglesias et al., 2001; Peschl et al., 2017). These findings have spurred researchers to investigate the role of MOG-IgG in CNS diseases. With the development of cell-based assays (CBAs), MOG-IgG has frequently been detected in serum samples from patients with anti- aquaporin-4-antibody (AQP4-Ab)-seronegative neuromyelitis optica spectrum disorder (NMOSD). These patients frequently present with the clinical phenotypes, such as acute disseminated encephalomyelitis (ADEM), optic neuritis (ON), myelitis, brainstem encephalitis, cerebellar encephalitis, and cortical encephalitis (CE). These clinical syndromes associated with MOG-IgG are collectively referred to as MOG antibody-associated disease (MOGAD)(Banwell et al., 2023).

MOGAD stands as an inflammatory demyelinating disease of the CNS, marking a distinct clinical entity from NMOSD and multiple sclerosis (MS), with a single or recurrent course (Jarius et al., 2018; López-Chiriboga et al., 2018). Autoimmune thyroid disease (AITD) represents one of the most prevalent organ-specific autoimmune diseases, caused by immune system dysregulation that leads to in an immune attack on the thyroid (Antonelli et al., 2015). Previous studies have confirmed that AITD usually co-exists with NMOSD and MS, and delving into the significance of anti-thyroid antibodies (ATAbs) and thyroid function among these patient groups (Niederwieser et al., 2003; Munteis et al., 2007; Wang et al., 2016). Nonetheless, the exploration of thyroid function and ATAbs in MOGAD patients has been scant.

This study was designed to assess whether there were abnormalities in thyroid function or antibodies in adult patients with MOGAD and to unearth the clinical significance of thyroid dysfunction/ATAbs-seropositive in adult patients with MOGAD.

## 2 Materials and methods

### 2.1 Participants

The medical records of 36 adult inpatients diagnosed firstly with MOGAD between August 2019 and June 2023 in the Department of Neurology, Neuroimmune Center, the Affiliated Brain Hospital of Nanjing Medical University were meticulously reviewed. All inpatients fulfilled the criteria proposed by 2023 International MOGAD Panel. Exclusion criteria: (1) age <16 years old; (2) cerebrospinal fluid (CSF) or serum positive for anti-NMDAR antibodies; (3) a diagnosis of other autoimmune diseases or thyroid diseases prior to MOGAD diagnosis; (4) Individuals with a history of prior attacks.

In addition, sex- and age- matched healthy controls (*n* = 47) were identified from a large of subjects who underwent physical examinations at our hospital. These healthy controls had no known comorbidities and had not received any treatment before physical examination.

Clinical data primarily included gender, age at onset, follow-up years, symptoms of CNS demyelinating during attack (Meningoencephalitis, Brainstem encephalitis, CE, Transverse myelitis (TM), ON and other special types), attack sites, annual relapse rate (ARR), expanded disability status scale (EDSS) scores at blood sampling, serum/CSF MOG-IgG titer, ATAbs and thyroid function in serum, electroencephalogram (EEG), and magnetic resonance imaging (MRI). Thyroid function tests were typically completed within 24 h of admission, while lumbar puncture, MRI, and EEG were performed within 48 h of admission. The ARR was calculated based on the number of relapses during follow-up period until July 2023. All clinical data was collected prior to the use of corticosteroids and immunosuppressants, with the exception for ARR and follow-up years.

### 2.2 Laboratory examinations

The thyroid function indexes of MOGAD patients during attack and healthy controls were detected by magnetic antibody enzyme linked immunosorbent assay (MAIA), including anti-thyroglobulin antibody (TGAb), anti-thyroid peroxidase antibody (TPOAb), triiodothyronine (T3; reference range, 1.3–3.1 nmol/L), free triiodothyronine (FT3; reference range, 3.1–6.8 pmol/L), thyroxine (T4; reference range, 66–181 nmol/L), free thyroxine (FT4; reference range, 12-22 pmol/L), thyroid stimulating hormone (TSH; reference range, 0.27–4.2 mIU/L). TPO-Ab and TG-Ab were determined as positive or negative according to the laboratory testing protocols. Additionally, serum and cerebrospinal fluid samples from MOGAD patients were analyzed to identify MOG-IgG expression using CBAs.

### 2.3 MRI

All patients underwent MRI screening using the GE 3.0T MR scanner (Verio, Siemens AG, Munich, Germany). The MRI scan sequence includes T1 with or without gadolinium-DTPA enhancement, T2, and fluid-attenuated inversion recovery (FLAIR). Spinal cord MRI examinations were performed on patients with suspected myelopathy. MRI images were analyzed and lesion locations were recorded by experienced radiologists who were blind to diagnostic and clinical information. Brain lesions of MOGAD patients are categorized into typical and atypical lesions based on established literature references (Ambrosius et al., 2020; Shahriari et al., 2021; Sechi et al., 2022).

### 2.4 EEG

The EEG was placed according to the 10/20 international system and at least 16 EEG channels were performed using a video electroencephalographer (EEG-9200K, NIHON KOHDEN, Japan) with hyperventilation and photostimulation. EEG reports were written by an eminent neurologist.

### 2.5 Assessment of dysfunction

Thyroid dysfunction was diagnosed according to the 2018 International Guidelines on Thyroid Function Testing in the Diagnosis and Monitoring of Thyroid Dysfunction. The EDSS scores were utilized to assess the severity of disability in MOGAD patients, with EDSS scores > 3 being indicative of an unfavorable condition.

### 2.6 Statistical analysis

Statistical analysis was conducted using SPSS (version 26.0, Chicago, America). A *P* value of <0.05 was considered statistically significant. Continuous variables were represented by the mean ± standard deviation (SD), with an independent sample *t*-test for those conforming to normal distribution and Wilcoxon Mann–Whitney U test for those not conforming to the normal distribution. Categorical variables were expressed by n (%), and analyzed using Chi-square test or Fisher exact test. The Spearman correlation analysis was used to analyze the correlation between thyroid hormones and EDSS scores.

## 3 Result

### 3.1 Demographic characteristics and results of thyroid function tests in patients with MOGAD and healthy controls

A total of 36 patients initially diagnosed with MOGAD were enrolled in this study. The demographic characteristics and thyroid function metrics of MOGAD patients and healthy controls are detailed in Table 1. There was no significant difference in gender distribution or age at onset between the two groups (18:18 vs. 26:21, *p* = 0.663; 35.25 ± 17.41 vs. 38.83 ± 12.02, *p* = 0.271). FT3, TT3, and FT4 levels were significantly lower in MOGAD patients compared to healthy controls (*p* < 0.0001, *p* < 0.0001, *p* = 0.03), although TSH levels and the abnormal ATAbs positivity did not show notable variance between the groups (*p* = 0.164, *p* = 0.520). Besides, the TT4 levels in MOGAD patients were lower than in healthy controls, but the difference was not statistically significant.

**Table 1:** Demographic and thyroid function examinations in patients with MOGAD and healthy controls.

	**MOGAD (*n* = 36)**	**HC group (*n* = 47)**	***P* value**
Gender F:M	18:18	26:21	0.663
Age at onset, year	35.25 ± 17.41	38.83 ± 12.02	0.271
**Thyroid hormone**			
TT3 (nmol/L)	1.33 ± 0.38	1.79 ± 0.37	**<0.0001**
FT3 (pmol/L)	3.73 ± 0.97	4.81 ± 0.73	**<0.0001**
TT4 (nmol/L)	86.76 ± 19.67	117.09 ± 136.03	0.189
FT4 (pmol/L)	15.90 ± 2.83	16.72 ± 2.78	**0.03**
TSH (mIU/L)	2.78 ± 5.41	2.40 ± 1.50	0.164
ATAbs	6 (16.7%)	5 (10.6%)	0.520
TPOAb (+)	5 (13.9%)	5 (10.6%)	0.740
TGAb (+)	5 (13.9%)	1 (2.1%)	0.081

MOGAD, myelin oligodendrocyte glycoprotein antibody-associated disease; HC group, healthy controls group; F, female; M, male; TSH, thyroid-stimulating hormone; FT3, free triiodothyronine; TT3, total triiodothyronine; FT4, free thyroxin; TT4, total thyroxin; TATbs, anti-thyroid antibodies; TPOAb, anti-thyroid peroxidase antibodies; TGAb, anti-thyroglobulin antibodies.

The value in bold indicates that the value <0.05, which is statistically significant.

### 3.2 Prevalence and distribution of thyroid dysfunction in patients with MOGAD and healthy controls

In our study, a significantly higher proportion of thyroid dysfunction was observed in patients with MOGAD than in healthy controls (*p* < 0.0001), with low T3 syndrome being the most frequently and statistically significant (*p* = 0.03) (Table 2). The incidence of subclinical hypothyroidism (*p* = 0.396) and clinical hypothyroidism (*p* = 1.0) was not significantly different between the two groups.

**Table 2:** Prevalence of thyroid dysfunction in patients with MOGAD and healthy controls.

	**MOGAD (*n* = 36)**	**HC group (*n* = 47)**	***P* value**
Thyroid dysfunction	20 (55.6%)	7 (14.9%)	**<0.0001**
Clinical hypothyroidism	4 (11.1%)	2 (4.3%)	0.396
Subclinical hypothyroidism	3 (8.3%)	3 (6.4%)	1.000
Low T3 syndrome	9 (25%)	1 (2.1%)	**0.002**

MOGAD, myelin oligodendrocyte glycoprotein antibody-associated disease; HC group, healthy controls group.

The value in bold indicates that the value <0.05, which is statistically significant.

### 3.3 Comparison of demographic and clinical features of MOGAD patients with and without low T3 syndrome

**Figure 1:**
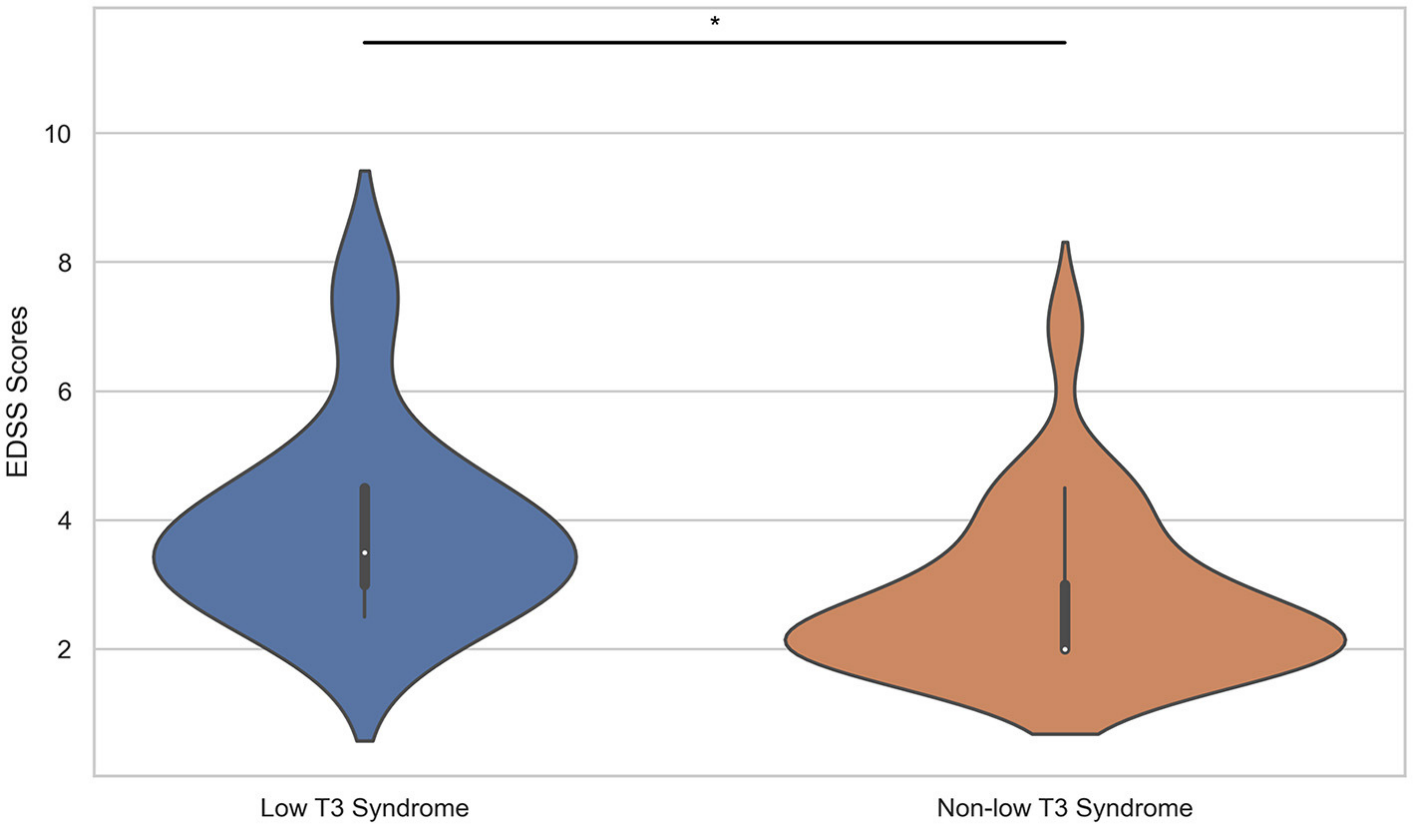
Comparison of EDSS scores between the low T3 syndrome group and the non-low T3 syndrome group. EDSS, expanded disability status scale; T3, triiodothyronine. *P < 0.05.

MOGAD patients were divided into two groups: the low T3 syndrome group and the non-low T3 syndrome group. The demographic and clinical features of both groups were summarized in Table 3. The median follow-up duration was 2.60 years for the low T3 syndrome group and 2.40 years for the non-low T3 syndrome group, with no significant differences in the ARR. There were also no significant differences between the two groups in sex ratio, age at onset, or serum/cerebrospinal fluid antibody titers. A higher occurrence of clinical phenotypes such as ON, TM, and brainstem encephalitis was noted in the low T3 syndrome group. Interestingly, none of the five patients who developed cortical encephalitis exhibited low T3 syndrome, although this finding was not statistically significant. The mean EDSS scores in the low T3 syndrome group were significantly higher compared to those without it (*p* = 0.014), with a significantly higher proportion of patients in the former group having EDSS scores >3 (*p* = 0.046, Figure 1).

**Table 3:** Demographic and clinical features between patients with and without low T3 syndrome.

	**Patients with MOGAD (*****n*** = **36)**		***P* value**
	**low T3 syndrome group (*****n*** = **9)**	**Non-low T3 syndrome group (*****n*** = **27)**	
Gender F:M	6:4	38:35	0.743
Age at onset	43.30 ± 21.41	36.45 ± 13.43	0.166
Follow-up years	2.6(0.25, 3.50)	2.40 (0.58, 3.83)	0.898
**-Lg(antibody titer)**			
Serum	1.45 ± 1.02	1.24 ± 1.10	0.598
CSF	0.17 ± 0.35	0.67 ± 0.80	0.097
ARR	0.56 ± 0.38	0.45 ± 0.42	0.415
EDSS scores	3.94 ± 1.49	2.89 ± 1.24	**0.014**
EDSS scores>3	6 (66.7%)	7 (25.9%)	**0.046**
**Clinical syndrome**			
Optic neuritis	4 (44.4%)	5 (18.5%)	0.184
Meningoencephalitis	5 (55.6%)	17 (63.0%)	0.712
Brainstem encephalitis	3 (33.3%)	7 (25.9%)	0.686
Cortical encephalitis	0	5 (18.5%)	0.302
Transverse myelitis	4 (44.4%)	7 (25.9%)	0.409

MOGAD, myelin oligodendrocyte glycoprotein antibody-associated disease; T3, triiodothyronine; EDSS, expanded disability status scale; CSF, cerebrospinal fluid; ARR, annual replase rate.

The value in bold indicates that the value <0.05, which is statistically significant.

There were 6 patients with EDSS scores > 3 in the low T3 syndrome group. A further analysis of their clinical phenotypes is provided in Supplementary Table 1, showing that meningoencephalitis constituted 50% of cases, followed by optic neuritis, brainstem encephalitis, and transverse myelitis, each accounting for 33%.

### 3.4 EEG and MRI findings between MOGAD patients with and without low T3 syndrome

We performed a comparative analysis between the two groups in terms of EEG abnormalities, specific location of the lesion, and segment of the spinal lesion, as shown in Table 4. These differences didn't achieve statistical significance.

**Table 4:** MRI findings and EEG examinations between patients with and without low T3 syndrome.

	**Patients with MOGAD (*****n*** = **36)**		***P* value**
	**With low T3 syndrome (*****n*** = **9)**	**Without low T3 syndrome (*****n*** = **27)**	
EEG abnormalities	3 (33.3%)	13 (48.1%)	0.700
**Brain lesion**			
MOGAD-typical brain lesion	8 (88.9%)	20 (74.1%)	0.648
Lateral ventricle	5 (55.6%)	10 (37.0%)	0.443
Cortical and subcortical	6 (66.7%)	12 (44.4%)	0.443
Thalamus	2 (22.2%)	4 (14.8%)	0.627
MOGAD-atypical brain lesion	4 (44.4%)	12 (44.4%)	1.000
Corpus callosum	2 (22.2%)	2 (7.4%)	0.255
Capsule	0 (0.00%)	1 (18.5%)	1.000
Brainstem	3 (33.3%)	10 (37.0%)	1.000
Cerebellum	0 (0.00%)	2 (7.4%)	1.000
Spinal cord	2 (22.2%)	6 (22.2%)	1.000
Spinal cord>3	2 (22.2%)	3 (11.1%)	0.518
Optical nerve	4 (44.4%)	5 (18.5%)	0.184

EEG, electroencephalogram; MOGAD, myelin oligodendrocyte glycoprotein antibody-associated disease; T3, triiodothyronine.

### 3.5 Correlation between thyroid hormone levels and EDSS scores in patients with MOGAD

**Figure 2:**
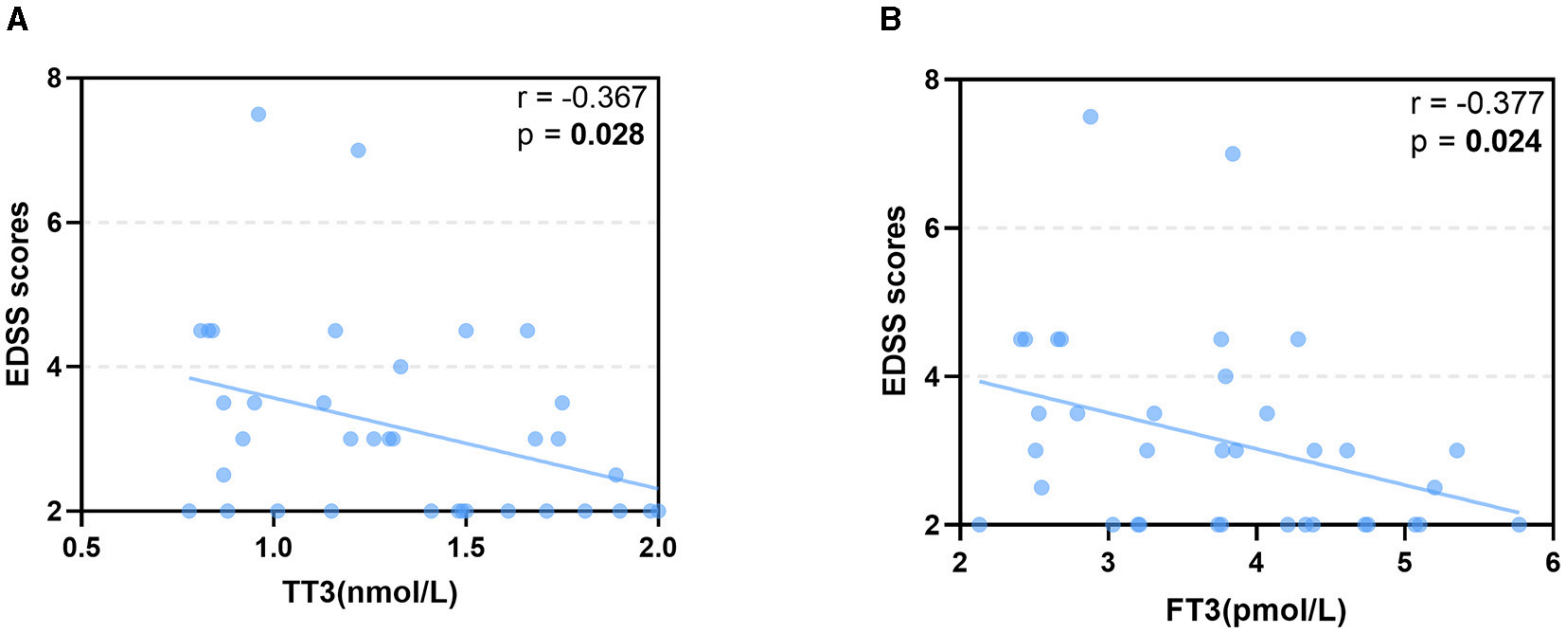
Spearman correlation was used to analyze the correlation between thyroid hormone levels and EDSS scores. The **(A, B)** show the negative significant association of TT3 and FT3 with EDSS. TSH, thyroid-stimulating hormone; FT3, free triiodothyronine; TT3, total triiodothyronine; EDSS, expanded disability status scale.

Spearman's correlation analysis was used to analyze the correlation between thyroid hormone levels and EDSS scores, as detailed in Table 5. A significant negative correlation was found between both TT3 and FT3 levels and EDSS scores (r = −0.367, *p* = 0.028, Figure 2A; r = −0.377, *p* = 0.024, Figure 2B), indicating that as T3 levels decreased, disability severity increased. However, TT4, FT4, and TSH levels showed no significant correlation with EDSS scores.

**Table 5:** Correlation between thyroid hormone levels and EDSS scores and ARR in patients with MOGAD.

	**EDSS scores**	
	**Correlation coefficient**	***P*** **value**
TT3	−0.367	**0.028**
TT4	−0.238	0.163
FT3	−0.377	**0.024**
FT4	−0.220	0.204
TSH	−0.112	0.515

MOGAD, myelin oligodendrocyte glycoprotein antibody-associated disease; TSH, thyroid-stimulating hormone; FT3, free triiodothyronine; TT3, total triiodothyronine; FT4, free thyroxin; TT4, total thyroxin; EDSS, expanded disability status scale.

The value in bold indicates that the value <0.05, which is statistically significant.

### 3.6 TATbs in patients with MOGAD

We further compared MOGAD patients with and without TG/TPO antibody double/single positivity in key aspects (Table 6). Except that the proportion of MOGAD-typical brain lesions and paralateral ventricle lesions was significantly lower in the TG/TPO antibody double/single positive group, there were no significant differences between the two groups concerning demography, clinical phenotype, results of laboratory tests, MRI findings, and EEG abnormalities.

**Table 6:** Comparison of main aspects between MOGAD patients with and without TG/TPO antibody double or single positive.

	**TG/TPO antibody double/single positive group (*n* = 6)**	**TG/TPO antibody double negative group (*n* = 30)**	***P* value**
Gender F:M	4:2	14:16	0.658
Age at onset	32.64 ± 16.65	37.99 ± 14.27	0.298
**-Lg (antibody titer)**			
Serum	0.92 ± 1.20	1.37 ± 1.04	0.293
CSF	1.00 ± 0.94	0.45 ± 0.66	0.134
EDSS scores	3.25 ± 1.13	3.08 ± 1.40	0.508
EDSS scores>3	3 (50.0%)	9 (30.0%)	0.378
**Clinical syndrome**			
Optic neuritis	3 (50.0%)	6 (20.0%)	0.184
Meningoencephalitis	2 (33.3%)	20 (66.7%)	0.181
Brainstem encephalitis	2 (33.3%)	8 (26.7%)	1.000
Cortical encephalitis	1 (16.7%)	4 (13.3%)	1.000
Transverse myelitis	1 (16.7%)	10 (33.3%)	0.643
EEG abnormalities	2(33.3%)	14 (46.7%)	0.672
**Brain lesion**			
MOGAD-typical brain lesion	1 (16.7%)	27 (90.0%)	**0.001**
Para lateral ventricle	0	15 (50.0%)	**0.03**
Cortical and subcortical	1 (16.7%)	17 (56.7%)	0.177
Thalamus	2 (22.2%)	4 (14.8%)	0.627
MOGAD-atypical brain lesion	2 (33.3%)	14 (46.7%)	0.672
Corpus callosum	0	4 (13.3%)	1.000
Capsule	1 (2.8%)	0	0.167
Brainstem	2 (33.3%)	11 (36.7%)	1.000
Cerebellum	0 (0.00%)	2 (6.7%)	1.000
Spinal cord	1 (16.7%)	7 (23.3%)	1.000
Spinal cord>3	1 (16.7%)	4 (13.3%)	1.000
Optical nerve	3 (50.0%)	5 (18.5%)	0.151

MOGAD, myelin oligodendrocyte glycoprotein antibody-associated disease; F:M, female: male; EEG, electroencephalogram; CSF, cerebrospinal fluid; EDSS, expanded disability status scale. TPO, anti-thyroid peroxidase; TG, anti-thyroglobulin.

The value in bold indicates that the value <0.05, which is statistically significant.

## 4 Discussion

Our research is the first to underscore the significance of thyroid function and antibodies in adult patients with MOGAD. Notably, thyroid dysfunction, particularly low T3 syndrome, occurred with greater frequency in MOGAD patients compared to healthy controls. Moreover, our findings revealed that EDSS scores differed significantly between MOGAD patients with and without low T3 syndrome. In addition, we observed that ATAbs-positive patients had fewer typical brain damage and para-lateral ventricle damage compared with ATAbs-negative patients, even though the seroprevalence of ATAbs was not statistically different between the MOGAD patients and healthy controls.

Some researchers have shown that low T3 syndrome is often present in various autoimmune diseases, such as systemic lupus erythematosus, MS, and NMOSD (Niederwieser et al., 2003; Antonelli et al., 2010; Kumar et al., 2012; Cho et al., 2016; Carnero Contentti et al., 2023). Cho et al. (2016) reported that 56.9% NMOSD patients had thyroid dysfunction and the most prevalent type is low T3 syndrome, accounting for 22.4%, followed by subclinical hyperthyroidism, subclinical hypothyroidism, and clinical hypothyroidism. Consistent with obvious studies, thyroid dysfunction occurs in about half of adult MOGAD patients (55.6%) in this study, with low T3 syndrome occupying the highest proportion (25%).

It is traceable to a relatively higher incidence of low T3 syndrome in adult patients with MOGAD. Deiodinases (Ds) are dimeric integral membrane proteins involved in the activation and/or inactivation of thyroid hormone (TH), including type 1, type2, and type 3 (Gereben et al., 2008). Type 2 iodothyronine deiodinase (D2) is responsible for facilitating the conversion of T4 to T3. While type-3 iodothyronine deiodinase (D3) is the main physiological inactivator that promotes the conversion of T3 to T2 (Darras et al., 2015). The experimental model of MS, characterized by severe CNS inflammation, oligodendrocyte degeneration, and severe demyelination, showed elevated levels of D3 mRNA in inflammatory expression regions (D'Intino et al., 2011; Dell'Acqua et al., 2012). Similarly, studies in rats also confirmed that D3 expression increased rapidly in distal and proximal segments after nerve injury (Li et al., 2001). More than that, it had been reported that increased inflammatory cytokines and chemokines also contributed to D2 dysregulation and increased D3 activity (Calza et al., 2015). These factors collectively might underpin the susceptibility of MOGAD patients to low T3 syndrome. Although the specific pathogenic mechanism of MOGAD remain somewhat elusive, the brain biopsy results of one MOGAD patient showed that the inflammatory demyelination manifestations, in line with a type II MS pattern, included partial axon retention, reactive astrocyte scarring, T cell, macrophage, or microglia infiltration, and complement deposition (Jarius et al., 2016). These observations suggest that the reduction in serum T3 levels in MOGAD patients could primarily result from D2 inactivation and D3 activation. Another plausible explanation for low T3 syndrome involves the regulatory effect of IL-6 on TH, with IL-6 reported to inhibit TH activation while promoting its inactivation in human cells (Wajner et al., 2011). Despite the uncertain role of IL-6 in MOGAD to date, the disease's features, such as antibody- and complement-mediated CNS damage, increased IL-6 levels in CSF, and the efficacy of IL-6 inhibitors, underscore the involvement of IL-6 in the disease's pathogenesis (Kothur et al., 2016; Kaneko et al., 2018; Ringelstein et al., 2022).

Our study also found that patients with low T3 syndrome had higher mean EDSS scores and a higher proportion of unfavorable condition. Moreover, the correlation analysis showed a significantly negative correlation between TT3 and FT3 levels and EDSS scores in MOGAD patients, suggesting that low T3 syndrome is associated with the severity of MOGAD exacerbation during acute attacks. MOGAD is characterized by inflammatory demyelination (Marignier et al., 2021), and previous studies have highlighted the critical role of T3 in the development, maturation, and regeneration of myelin. For instance, the necessity of T3 for oligodendrogenesis and developmental myelination has been corroborated by the reduced CNS myelination observed in hypothyroid sufferers and rodents (Calza et al., 2015). Experimental work demonstrated that T3 acted on the formation of myelinating oligodendrocytes by stimulating oligodendrocyte progenitor cells to stop proliferating to induce cell cycle exit and terminal differentiation or other distinct molecular pathways both *in vivo* and *in vitro* (Calza et al., 2002; Raff, 2006). Furthermore, there are studies have suggested that T3 administration improves remyelination (Franco et al., 2008; Harsan et al., 2008). Based on these findings, we put forward the hypothesis that increased the exacerbation of disease observed in MOGAD patients with low T3 syndrome during the episode, possibly related to the critical function of T3 in the CNS. Therefore, it is necessary for first-line clinicians to timely identify and correct low T3 syndrome, but further clinical research is needed to determine whether this can greatly improve the disability of patients.

The prevalence of positive TPO-Ab and TG-Ab was reported in patients with NMOSD, ranging from 20.9% to 49% and 27.9% to 38.8%, respectively (Sellner et al., 2011; Long et al., 2014). Intriguingly, we found that TPO-Ab and TG-Ab positivity were less frequently observed in MOGAD patients (13.9%, 13.9%). What is noteworthy is that the existing studies have highlighted that a significant difference in the incidence of positive ATAbs between NMOSD patients and healthy subjects and indicated that a potential association between ATAbs status and various clinical outcomes, including disability status, EEG abnormalities, and longitudinally extensive spinal cord lesions (Li et al., 2015; Huo et al., 2022). However, our study did not replicate these findings. Typical brain lesions and paralateral ventricle lesions were significantly rare in patients with positive ATAbs compared to those with negative ATAbs, possibly due to the sparse number of patients with positive ATAbs. Therefore, large-scale studies are urgently required to clarify the clinically meaningful of these results.

There are some limitations to our study. Firstly, this was a single-center with small-sample size study and limited to adult Chinese. Inevitably, this leads to selection bias and limits the generalizability of our findings. Thus, the conclusion drawn needs further confirmation using large sample sizes and multi-center studies. Secondly, Furthermore, although CBAs are considered a relatively accurate method for detecting MOG-IgG at present, false positives can also occur, thereby potentially affecting the accuracy of antibody titers. Finally, our study only focused on thyroid dysfunction in MOGAD patients during acute attack and lacked clinical data on remission. Future studies should include data from the remission phase to determine whether the findings from the acute phase are applicable to the remission phase in MOGAD patients.

## 5 Conclusion

Overall, this study confirmed thyroid dysfunction is frequent in adult MOGAD patients, especially low T3 syndrome. Patients with low T3 syndrome had higher EDSS scores and a significantly higher proportion of unfavorable condition. Moreover, the correlation analysis model manifests that FT3 and TT3 levels were negatively correlated with the EDSS scores. These evidences indicate that low T3 syndrome is associated with the severity of MOGAD exacerbation.

## Data availability statement

The original contributions presented in the study are included in the article/Supplementary material, further inquiries can be directed to the corresponding author.

## Ethics statement

The studies involving humans were approved by the Ethics Committee of the Affiliated Brain Hospital of Nanjing Medical University. The studies were conducted in accordance with the local legislation and institutional requirements. The participants provided their written informed consent to participate in this study. Written informed consent was obtained from the individual(s) for the publication of any potentially identifiable images or data included in this article.

## Author contributions

YW: Conceptualization, Data curation, Formal analysis, Methodology, Writing – original draft, Writing – review & editing. HZ: Data curation, Formal analysis, Writing – review & editing. XC: Visualization, Writing – review & editing. JL: Funding acquisition, Supervision, Writing – review & editing.
