# Supplementary material for: Low T3 syndrome is associated with the severity of myelin oligodendrocyte glycoprotein antibody-associated disease exacerbation
Source: Front Neurosci. 2024 May 21;18:1357633. doi: 10.3389/fnins.2024.1357633 (PMC11148359; doi:10.3389/fnins.2024.1357633)
Supplement: Supplementary file 1 [file Table_1.DOCX]

**Table 1**

**Clinical phenotypes of 6 patients with EDSS scores >3 from the low T3 syndrome group**

| Clinical syndrome | Patient 1 | Patient 2 | Patient 3 | Patient 4 | Patient 5 | Patient 6 |
| --- | --- | --- | --- | --- | --- | --- |
| Optic neuritis |  |  | + |  |  | + |
| Meningoencephalitis |  | + |  | + | + |  |
| Brainstem encephalitis | + | + |  |  |  |  |
| Cortical encephalitis |  |  |  |  |  |  |
| Transverse myelitis | + |  |  |  | + |  |

EDSS, expanded disability status scale.
